# Supplementary material for: Analysis of PPARγ Signaling Activity in Psoriasis
Source: Int J Mol Sci. 2021 Aug 10;22(16):8603. doi: 10.3390/ijms22168603 (PMC8395241; doi:10.3390/ijms22168603)
Supplement: Supplementary file 1 [file ijms-22-08603-s001.zip › Supplemental materials_Analysis of PPARg signaling activity in psoriasis/Pathway models/Models images and html files/Model of PPARG signaling in psoriasis/13531.html]

APLN


# Protein APLN

|  |  |
| --- | --- |
| URN | urn:agi-llid:8862 |
| Total Entities | 3 |
| Connectivity | 2082 |
| Name | APLN |
| Description | apelin |
| Notes | This gene encodes a peptide that functions as an endogenous ligand for the G-protein coupled apelin receptor. The encoded preproprotein is proteolytically processed into biologically active C-terminal peptide fragments. These peptide fragments activate different tissue specific signaling pathways that regulate diverse biological functions including fluid homeostasis, cardiovascular function and insulin secretion. This protein also functions as a coreceptor for the human immunodeficiency virus 1. [provided by RefSeq, Feb 2016] |
| Primary Cell Localization | Extracellular |
| Class | Ligand |

---

|  |  |
| --- | --- |
| ChildConcepts | apelin-36 |
|  | apelin-17 |
|  | apelin-13 |

---

|  |  |
| --- | --- |
| Pathway | Adipokines Production by Adipocyte |
|  | Proteins Involved in Atherosclerosis |
|  | Proteins Involved in Dilated Cardiomyopathy |
|  | Proteins Involved in Arterial Hypertension |
|  | Proteins Involved in Pulmonary Hypertension |
|  | Proteins Involved in Myocardial Ischemia |
|  | Proteins Involved in Polycystic Ovary Syndrome |
|  | Proteins Involved in Epilepsy |
|  | Proteins Involved in Amyotrophic Lateral Sclerosis |
|  | GH1/GHR -> STAT Expression Targets |
|  | GH1/PRLR Expression Targets |
|  | Model of PPARG signaling in psoriasis |
|  | drug-target moltransport |

---

|  |  |
| --- | --- |
| Group | Genes with Mutations Associated with Obesity |

---

|  |  |
| --- | --- |
| MedScan ID | 8862 |

---

|  |  |
| --- | --- |
| LocusLink ID | 8862 |
|  | 30878 |
|  | 58812 |

---

|  |  |
| --- | --- |
| Alias | peptide ligand for APJ receptor |
|  | peptide ligand for APJ receptors |
|  | AGTRL1 ligand |
|  | RP23-455F14.1 |
|  | 6030430G11 |
|  | APLN |
|  | 6030430G11Rik |
|  | APJ endogenous ligand |
|  | UNQ471 |
|  | apelin, peptide ligand for APJ receptor |
|  | apelin, AGTRL1 ligand |
|  | apelin, peptide ligand for APJ receptors |
|  | Apel |
|  | apelin |
|  | OTTMUSP00000019097 |
|  | RIKEN cDNA 6030430G11 gene |
|  | preproapelin |
|  | XNPEP2 |

---

|  |  |
| --- | --- |
| GO ID | 0031704 |
|  | 0005179 |
|  | 0042803 |
|  | 0005102 |
|  | 0007186 |
|  | 0001525 |
|  | 0060183 |
|  | 0060976 |
|  | 0042756 |
|  | 0007369 |
|  | 0006955 |
|  | 0007595 |
|  | 0045776 |
|  | 0040037 |
|  | 0010629 |
|  | 1904706 |
|  | 0045906 |
|  | 1904022 |
|  | 0051461 |
|  | 0051466 |
|  | 0045823 |
|  | 0031652 |
|  | 0042327 |
|  | 1902895 |
|  | 1905564 |
|  | 0043576 |
|  | 0002026 |
|  | 0007165 |
|  | 0005576 |
|  | 0005615 |
|  | 0048471 |
|  | 0007631 |
|  | 0007275 |
|  | 0097755 |
|  | 0008284 |
|  | 0050878 |
|  | 0010469 |

---

|  |  |
| --- | --- |
| KEGG ID | hsa:8862 |
|  | mmu:30878 |
|  | rno:58812 |

---

|  |  |
| --- | --- |
| Organism | Homo sapiens {Organism urn:agi-taxid:9606} |
|  | Mus musculus {Organism urn:agi-taxid:10090} |
|  | Rattus norvegicus {Organism urn:agi-taxid:10116} |
|  | Homo sapiens |
|  | Mus musculus |
|  | Rattus norvegicus |

---

|  |  |
| --- | --- |
| Mouse chromosome position | X |

---

|  |  |
| --- | --- |
| OMIM ID | 300297 |

---

|  |  |
| --- | --- |
| Rat chromosome position | Xq36 |

---

|  |  |
| --- | --- |
| Hugo ID | 16665 |

---

|  |  |
| --- | --- |
| Human chromosome position | Xq26.1 |

---

|  |  |
| --- | --- |
| Swiss-Prot Accession | Q9ULZ1 |
|  | Q9ULZ1.1 |
|  | Q544B5 |
|  | Q9R0R4 |
|  | Q9R0R4.1 |
|  | Q9R0R3 |
|  | Q9R0R3.1 |
|  | Q4VY08 |
|  | Q8WU89 |

---

|  |  |
| --- | --- |
| GenBank ID | NC\_000023 |
|  | NM\_017413 |
|  | NP\_059109 |
|  | NG\_016718 |
|  | AF179680 |
|  | AAF25815 |
|  | AL022162 |
|  | CH471107 |
|  | EAX11828 |
|  | EAX11829 |
|  | DQ385612 |
|  | AB023493 |
|  | BAA84975 |
|  | AF086248 |
|  | AK001855 |
|  | AK312061 |
|  | BAG34997 |
|  | AY358657 |
|  | AAQ89020 |
|  | BC021104 |
|  | AAH21104 |
|  | BM992527 |
|  | DB481574 |
|  | Q9ULZ1 |
|  | NC\_000086 |
|  | NM\_013912 |
|  | NP\_038940 |
|  | AL672274 |
|  | AL714010 |
|  | CH466570 |
|  | EDL29063 |
|  | AB023494 |
|  | BAA84976 |
|  | AJ290423 |
|  | CAC83678 |
|  | AK020057 |
|  | AK042076 |
|  | BAC31156 |
|  | BB618863 |
|  | BC020015 |
|  | AAH20015 |
|  | DQ832282 |
|  | ABI30216 |
|  | Q9R0R4 |
|  | NC\_005120 |
|  | NM\_031612 |
|  | NP\_113800 |
|  | AC\_000089 |
|  | AAHX01114444 |
|  | AC127934 |
|  | CH473991 |
|  | EDM10904 |
|  | AB023495 |
|  | BAA84977 |
|  | AF179679 |
|  | AAF25814 |
|  | BC080843 |
|  | AAH80843 |
|  | Q9R0R3 |

---

|  |  |
| --- | --- |
| Swiss-Prot ID | APEL\_HUMAN |
|  | APEL\_MOUSE |
|  | APEL\_RAT |

---

|  |  |
| --- | --- |
| Cell Localization | Secreted |
|  | Extracellular space |

---

|  |  |
| --- | --- |
| Ensembl ID | ENSG00000171388 |
|  | ENSP00000391800.2 |
|  | ENST00000429967.3 |
|  | ENSMUSG00000037010 |
|  | ENSMUSP00000046012.7 |
|  | ENSMUST00000039026.7 |
|  | ENSRNOG00000003984 |
|  | ENSRNOP00000005331.3 |
|  | ENSRNOT00000005331.5 |

---

|  |  |
| --- | --- |
| MGI ID | MGI:1353624 |

---

|  |  |
| --- | --- |
| RGD ID | 620672 |

---
